# Lebanese Waterpipe Dependence Scale (LWDS-11) validation in a sample of Lebanese adolescents

**DOI:** 10.1186/s12889-021-11662-3

**Published:** 2021-09-06

**Authors:** Souheil Hallit, Sahar Obeid, Hala Sacre, Pascale Salameh

**Affiliations:** 1 Faculty of Medicine and Medical Sciences, Holy Spirit University of Kaslik (USEK), Jounieh, Lebanon; 2 INSPECT-LB: Institut National de Santé Publique, Épidémiologie Clinique et Toxicologie-Liban, Beirut, Lebanon; 3 Research Department, Psychiatric Hospital of the Cross, Jal Eddib, Lebanon; 4 Faculty of Arts and Sciences, Holy Spirit University of Kaslik (USEK), Jounieh, Lebanon; 5 Department of Primary Care and Population Health, University of Nicosia Medical School, Nicosia, Cyprus; 6 Faculty of Pharmacy, Lebanese University, Beirut, Lebanon

**Keywords:** Waterpipe, Dependence, LWDS, Adolescents, Lebanon

## Abstract

**Background:**

Salameh et al. developed the Lebanese Waterpipe Dependence Scale (LWDS-11) that assesses nicotine dependence among adult waterpipe smokers. In view of the high waterpipe use among Lebanese youth and other neighboring countries, it was deemed necessary to check the psychometric properties of the LWDS-11, originally adapted to the Lebanese population, to measure nicotine dependence among adolescents.

**Methods:**

Two cross-sectional investigations were conducted; Study 1 (January and May 2019) enrolled a total of 449 students who were exclusive waterpipe smokers; this sample was used to conduct the exploratory factor analysis. Study 2 enrolled another sample composed of 243 waterpipe smoking adolescents. This sample was independent from the first one and was used to conduct the confirmatory analysis.

**Results:**

The results also showed that 312 (69.5%) [95% CI 0.652–0.738] had high waterpipe dependence (scores of ≥10). Results of the factor analysis in sample 1 showed that all LWDS-11 items were extracted following the factor analysis. Items converged over a solution of one factor; total variance explained = 70.45%, α_Cronbach_ = 0.96). The results of the confirmatory factor analysis were as follows: the Maximum Likelihood Chi-Square = 129.58 and Degrees of Freedom = 45, which gave a χ^2^/df = 2.88. For non-centrality fit indices, the Steiger-Lind Root Mean Square Error of Approximation (RMSEA) was 0.08 [0.071–0.106]. Moreover, the Comparative Fit Index (CFI) value was 0.77.

**Conclusion:**

The preliminary results suggest that the LWDS-11 has good psychometric properties to measure waterpipe dependence among adolescents. We hope this tool would serve the benefit of research and epidemiology.

## Background

Waterpipe smoking (WPS) is gaining more popularity worldwide among adolescents, with a prevalence varying from 6 to 34% [1]. Higher tendencies of WPS were reported in Middle Eastern countries in general, [2] and in Lebanon specifically (36.9% among youth between 13 and 15 years) [3]. This might be because WPS among adolescents is more culturally acceptable than cigarette smoking and might also be shared with a family member [4]. The popularity of WPS is somewhat credited to a general misconception that it is less harmful than cigarettes [5–8]. Similar misconceptions have changed parents’ attitudes towards accepting WPS in adolescents compared to cigarettes [9]. Waterpipe smoking was linked to higher lipoproteins levels alone or in combination with cigarettes [10, 11] and risky behaviors (alcohol, smoking, sexual relationship without condom use, driving without seat belt, etc.) [12] in adults.

The Diagnostic Statistical Manual of Mental Disorders, fifth edition (DSM 5) and the International Classification of Diseases, 10th Revision (ICD-10) criteria for smoking dependence/addiction are similar for adults and adolescents and include: 1) Bigger amounts of tobacco are consumed over a longer time than intended; 2) tolerance for nicotine; 3) withdrawal symptoms when stopping smoking; and 4) diminished control over use and continuous relapses after attempting to quit [13]. While waterpipe dependence has been identified among adults [14], evolving evidence suggest that persons like adolescents, who smoked waterpipe for a short period, will also show symptoms of nicotine dependence [15]. The most frequently described symptoms of nicotine dependence include craving, feeling of addiction, and failure to quit, which were correlated to more waterpipe smoking in terms of frequency, number and length of waterpipe smoking sessions [16]. Symptoms of nicotine dependence due to waterpipe appear at a significantly lower number (7.5 waterpipes compared to 27.5 cigarettes per month) and frequency of use (6 vs 13.5 days per month) compared to cigarette smoking [17]. However, to the best of our knowledge, two scales only, the modified Waterpipe Tolerance Questionnaire- Arabic version [18] and the Syrian Center for Tobacco Studies-13 (SCTS-13) [15], were developed to measure nicotine dependence levels in WPS adolescents.

Salameh et al. [19] developed the Lebanese Waterpipe Dependence Scale (LWDS-11) that assesses nicotine dependence among adult WPS. This tool utilizes the symptoms of nicotine dependence from cigarette smoking described in the literature, although ND symptoms among waterpipe smokers might have different features (such as responsiveness to social and sensory cues). In view of the high WP use among Lebanese youth and other neighboring countries [18, 20, 21], it was deemed necessary to check the psychometric properties of the LWDS-11, originally adapted to the Lebanese population, to measure nicotine dependence among adolescents.

## Methods

### Participants and procedure

This cross-sectional investigation took place between January and May 2019. A list of schools was obtained from the Ministry of Education and Higher Education. A sample of schools from all Lebanese districts was chosen in a random and proportionate manner. Eighteen private schools were contacted, 2 refused to participate as they believed it would take too much time for the students to fill and they didn’t want to waste their time in class. The 16 schools were distributed as such: 4 in Beirut, 2 in South Lebanon, 6 in Mount Lebanon, 2 in North Lebanon, and 2 in Bekaa. No public schools were selected during this process. All students aged 12 to 17 years from each school were allowed to participate. They had the ability to agree or decline enrollment in the study, with no monetary payment in exchange for their involvement. Excluded were students that declined to fill the survey. This paper shares the same methodology as other papers from the same project [22–27].

### Minimal sample size

A minimum of ten participants per scale item is needed according to Comrey and Lee [28]. Since the LWDS-11 scale contains 11 questions, a minimal (theoretical) sample of 110 participants was deemed necessary in order to run an exploratory factor analysis.

Study 1: A total of 1810 (90.5%) out of the 2000 questionnaires distributed was collected back; 449 students (24.8%) were exclusive waterpipe smokers.

Study 2: Another sample, independent from the first one and composed of 243 adolescents (waterpipe smokers as well), was used to conduct the confirmatory analysis.

### Measures

The form used was in Arabic, and required approximately 10 min to be completed. Students were requested to fill out the form at school to avoid any influence from their parents. Participants’ anonymity was guaranteed throughout the information gathering procedure.

The first part of the questionnaire measured students’ sociodemographic information (age, sex, smoking status). Participants were divided into four groups: nonsmokers, exclusive cigarette smokers, exclusive waterpipe smokers, and dual (waterpipe and cigarette) smokers. Current WPS was defined as smoking ≥1 waterpipe per week [29], whereas current cigarette smoking was defined as smoking at least one cigarette in the last 30 days [30]. The later part of the survey included the Lebanon Waterpipe Dependence Scale-11 (LWDS-11), used to assess waterpipe dependence [19]. It includes 11 items measured on a 4-point Likert scale ranging from 0 to 3. High waterpipe dependence was defined as having a score of LWDS-11 ≥ 10 [19] (α_Cronbach_ in this study = 0.888).

### Statistical analysis

We did not replace/impute missing data since it constituted < 10% of the full database. Reliability was checked using Cronbach’s alpha for the total scale and its subscales. Using the FACTOR software, a polychoric (tetrachoric) correlation was initiated using the “principal component analysis” technique of the LWDS-11 items in sample 1 among exclusive waterpipe smokers only. The parallel analysis determined the number of factors to retain. The Kaiser-Meyer-Olkin (KMO) value and the Bartlett’s sphericity test ensured sampling adequacy. Factors with Eigen values > 1 were kept. For each test item, the minimum cutoff value for factor loading was determined at 0.4.

SPSS AMOS v.24 software was used afterwards to conduct a confirmatory factor analysis on sample 2. Multiple indices of goodness-of-fit were described: the Relative Chi-square (χ2/df) (cut-off values:< 2–5), the Root Mean Square Error of Approximation (RMSEA) (close and acceptable fit are considered for values < 0.05 and < 0.11 respectively), the Comparative Fit Index (CFI) (acceptable values are ≥0.90) [31].

## Results

In sample 1, the mean age of the participants was 15.14 years (SD: 1.13), with 46.5% males. The results also showed that 312 (69.5%) [95% CI 0.652–0.738] had high waterpipe dependence (scores of ≥10). Other characteristics of both samples can be found in Table 1.

**Table 1:** Sociodemographic characteristics of the sample population

	Original sample (*N* = 1810)	Sample 1 (*N* = 449)	Sample 2 (*N* = 243)
**Sex**			
Male	844 (46.7%)	209 (46.5%)	70 (17.4%)
Female	963 (53.3%)	240 (53.5%)	333 (82.6%)
**Smoking status**			
Non-smokers	1342 (74.1%)	–	–
Exclusive cigarette smokers	395 (21.9%)	–	–
Exclusive waterpipe smokers	449 (24.9%)	449 (100%)	243 (100%)
Dual (cigarette and waterpipe) smokers	376 (20.8%)	–	–
	**Mean ± SD**	**Mean ± SD**	**Mean ± SD**
**Age (years)**	15.42 ± 1.14	15.14 ± 1.13	16.55 ± 0.97

### Exploratory factor analysis (EFA) among exclusive waterpipe smokers only in sample 1

All LWDS-11 items were extracted following the factor analysis. Items converged over a solution of one factor; total variance explained = 70.45%, KMO = 0.855 (good); Bartlett’s test of sphericity *p* < 0.001; α_Cronbach_ = 0.96). Correlation coefficients between each scale’s question and the total score ranged between 0.627 and 0.911 (p < 0.001 for all) (Table 2).

**Table 2:** Principal component analysis of the LWDS-11 items among waterpipe smokers only (*N* = 449)

LWDS-11 item	Factor 1	h2 communalities	Item-total correlation*
LWDS 1. Number of times you could stop waterpipe for 7 days?	0.881	0.776	0.884
LWDS 2. Percent of income you would spend on waterpipe smoking?	0.667	0.445	0.665
LWDS 3. Number of days you could spend without waterpipe?	0.806	0.649	0.815
LWDS 4. Number of water pipes you usually smoke per week?	0.622	0.387	0.627
LWDS 5. You smoke waterpipe to relax your nerves.	0.848	0.719	0.853
LWDS 6. You smoke waterpipe to improve your morale.	0.843	0.710	0.847
LWDS 7. Do you smoke waterpipe when you are seriously ill?	0.917	0.841	0.909
LWDS 8. Do you smoke waterpipe alone?	0.888	0.789	0.882
LWDS 9. Are you ready not to eat in exchange for a waterpipe?	0.898	0.806	0.890
LWDS 10. You smoke waterpipe for pleasure.	0.884	0.782	0.885
LWDS 11. You smoke to please others (for conviviality)	0.919	0.845	0.911
Percentage of variance explained	70.45		
Cronbach’s alpha	0.96		

*p < 0.001 for all correlations

### Confirmatory factor analysis among exclusive waterpipe smokers only in sample 2

A confirmatory factor analysis was run over another sample independent from the first one (sample 2; *N* = 243), using the one-factor solution obtained in the EFA. The results were as follows: the Maximum Likelihood Chi-Square = 129.58 and Degrees of Freedom = 45, which gave a χ^2^/df = 2.88. For non-centrality fit indices, the Steiger-Lind RMSEA was 0.08 [0.071–0.106]. Moreover, the CFI value was 0.77.

## Discussion

This study verified the psychometric properties of the originally-adapted-for-adults LWDS-11 scale to measure waterpipe dependence among Lebanese adolescents. Findings of the present paper suggest that the LWDS-11 has an excellent internal consistency (reliability) and evaluates waterpipe dependence in one single area, which includes psychological dependence (positive and negative reinforcement), physiological dependence (nicotine effect) and addictive aspect (money to be paid and potential duration of abstinence). The LWDS-11 questions were able to tackle all aspects of waterpipe dependence, including its social aspect, making this scale thorough in its assessment. Moreover, LWDS-11 items can be completed in less than 5 min, making it a very effective tool for research and practice to identify adolescents with waterpipe dependence.

### Factor analysis

Results of this study revealed that the LWDS-11 test items converged over a solution of one factor among exclusive waterpipe smokers. Those results are different than the ones obtained in the original validation of the LWDS among adults [19] where four dimensions were found: physiological nicotine dependence, termination of dysphoric states or negative reinforcement, psychological craving, and positive reinforcement (encompassing pleasure and social interaction). This leads us to underline that the concept of waterpipe dependence possibly varies between adolescents and adults. Further research are needed to understand those differences.

However, it is well known that the earlier smoking starts, the higher the chances of becoming addicted [32]. An experiment on tobacco use by adolescents considerably increases their risk of smoking in adulthood [33]. Physical and psychological dependence symptoms arise early after smoking initiation, with consequently higher consumption of tobacco [34]. Nicotine dependence can mark the transition from testing to regular smoking [35] and can increase the frequency of smoking as this transition occurs [36]. In addition, the social aspects of WPS attract adolescents, who recognize it as a stylish and nontoxic alternative to cigarettes, with lesser dependence potential [20]. In reality, findings reveal the exposure of WP smokers to big amounts of nicotine and exhibit nicotine-dependence symptoms similar to those experienced by cigarette smokers [14, 37]. Therefore, intervention and prevention strategies for waterpipe smoking must start early in adolescence and can have the ultimate influence by educating adolescents about the dangerous and addictive properties of WP, by coaching them about positive coping techniques, and addressing the use of waterpipes by family members and peers.

### Prevalence of waterpipe smokers in our sample

The percentage of adolescents with high waterpipe dependence (22.5% out of the total sample *N* = 1810 and 69.5% among waterpipe smokers) was higher than the prevalence of adolescents in Oman (9.6%) [21]. A systematic review [38] showed that the prevalence in the Middle Eastern countries was the highest, ranging from 12.9% in Iraqi adolescents in 2008 to 65.9% among Lebanese dents in Beirut (Lebanon) in 2002. The prevalence in Europe ranges from 12.0% in an English city to 49.5% in Swedish adolescents in 2011. Similar ranges were found in the United States (3.0–44.0%).

### Limitations

This study has few limitations. Information bias may be present because questions may not have been correctly understood. The study design is cross-sectional, thus, cannot assess the variation of the nicotine dependence symptoms through time. The use of a tool with answers following a Likert scale is likely to limit the variability of the items and may affect the factor structure. A selection bias is present since adolescents who do not attend schools were not approached and because of the schools selection process. A social desirability bias might also be present, since students tend to answer questions in a manner that will be positively viewed by others. No test-retest was done during the LWDS-11 scale. The CFA results were not very satisfactory; they were verified by the Maximum Likelihood Chi-Square value, borderline for the RMSEA value but below the minimum for the CFI value. Further studies are needed to assess the psychometric properties of the LWDS among adolescents. Finally, the results of this study cannot be generalized to the whole population since adolescents not enrolled in schools and those enrolled in public schools were excluded.

## Conclusion

The preliminary results of this study suggest that the LWDS-11 has good psychometric properties to measure waterpipe dependence among adolescents. We hope this tool would serve the benefit of research and epidemiology.

## Data Availability

The authors do not have the right to share any data information as per their institutions policies.

## Abbreviations

WP: Waterpipe
WPS: Waterpipe smoking
SCTS-13: Syrian Center for Tobacco Studies-13
LWDS-11: Lebanon Waterpipe Dependence Scale-11
KMO: Kaiser-Meyer-Olkin
RMSEA: Root Mean Square Error of Approximation
GFI: Goodness of fit index
AGFI: Adjusted goodness of fit index
DSM-5: Diagnostic Statistical Manual of Mental Disorders, fifth edition
ICD-10: International Classification of Diseases, 10th Revision

## References

[1] World Health Organization: Waterpipe Tobacco Smoking: Health Effects, Research Needs and Recommended Actions by Regulators. Available from: https://www.who.int/tobacco/global_interaction/tobreg/Waterpipe%20recommendation_Final.pdf.

[2] Al Suwaidi J, Zubaid M, El-Menyar AA, Singh R, Asaad N, Sulaiman K, Al Mahmeed W, Al-Shereiqi S, Akbar M, Al Binali HA (2012). Prevalence and outcome of cigarette and waterpipe smoking among patients with acute coronary syndrome in six middle-eastern countries. Eur J Prev Cardiol.

[3] Jawad M, Lee JT, Millett C (2016). Waterpipe tobacco smoking prevalence and correlates in 25 eastern Mediterranean and eastern European countries: cross-sectional analysis of the global youth tobacco survey. Nicotine Tob Res.

[4] Kheirallah KA, Alzyoud S, Ward KD (2015). Waterpipe use and cognitive susceptibility to cigarette smoking among never-cigarette smoking Jordanian youth: analysis of the 2009 global youth tobacco survey. Nicotine Tob Res.

[5] Aljarrah K, Ababneh ZQ, Al-Delaimy WK (2009). Perceptions of hookah smoking harmfulness: predictors and characteristics among current hookah users. Tob Induc Dis.

[6] Hallit S, Haddad C, Bou Malhab S, Khabbaz LR, Salameh P (2020). Construction and validation of the water pipe harm perception scale (WHPS-6) among the Lebanese population. Environ Sci Pollut Res Int.

[7] Wray RJ, Jupka K, Berman S, Zellin S, Vijaykumar S (2012). Young adults’ perceptions about established and emerging tobacco products: results from eight focus groups. Nicotine Tob Res.

[8] Jorge-Araujo P, Torres-García M, Marrero-Montelongo M, Navarro-Rodríguez C (2018). Beliefs and attitudes of spanish adolescents regarding waterpipe smoking. Enferm Glob.

[9] Jawaid A, Zafar AM, Rehman TU, Nazir MR, Ghafoor ZA, Afzal O, Khan JA (2008). Knowledge, attitudes and practice of university students regarding waterpipe smoking in Pakistan. Int J Tuberc Lung Dis.

[10] Hallit S, Hallit R, Haddad C, Youssef L, Zoghbi M, Costantine R, Kheir N, Salameh P (2019). Previous, current, and cumulative dose effect of waterpipe smoking on LDL and total cholesterol. Environ Sci Pollut Res Int.

[11] Hallit S, Zoghbi M, Hallit R, Youssef L, Costantine R, Kheir N, Salameh P (2017). Effect of exclusive cigarette smoking and in combination with waterpipe smoking on lipoproteins. J Epidemiol Glob Health.

[12] Hallit S, Sacre H, Salameh P (2020). Effect of waterpipe dependence on risk motives, attitudes and other health-related risky behaviors in Lebanese university students. Environ Sci Pollut Res Int.

[13] American Psychiatric Association (2013). Diagnostic and statistical manual of mental disorders: DSM-5.

[14] Aboaziza E, Eissenberg T (2015). Waterpipe tobacco smoking: what is the evidence that it supports nicotine/tobacco dependence?. Tob Control.

[15] Alam MM, Ward KD, Bahelah R, Kalan ME, Asfar T, Eissenberg T, Maziak W (2020). The Syrian Center for Tobacco Studies-13 (SCTS-13): psychometric evaluation of a waterpipe-specific nicotine dependence instrument. Drug Alcohol Depend.

[16] Bahelah R, DiFranza JR, Ward KD, Eissenberg T, Fouad FM, Taleb ZB, Jaber R, Maziak W (2017). Waterpipe smoking patterns and symptoms of nicotine dependence: the Waterpipe dependence in Lebanese youth study. Addict Behav.

[17] Bahelah R, DiFranza JR, Fouad FM, Ward KD, Eissenberg T, Maziak W (2016). Early symptoms of nicotine dependence among adolescent waterpipe smokers. Tob Control.

[18] Alzyoud S, Veeranki SP, Kheirallah KA, Shotar AM, Pbert L (2015). Validation of the Waterpipe tolerance questionnaire among Jordanian school-going adolescent Waterpipe users. Global J Health Sci.

[19] Salameh P, Waked M, Aoun Z (2008). Waterpipe smoking: construction and validation of the Lebanon Waterpipe Dependence Scale (LWDS-11). Nicotine Tob Res.

[20] Akl EA, Jawad M, Lam WY, Co CN, Obeid R, Irani J (2013). Motives, beliefs and attitudes towards waterpipe tobacco smoking: a systematic review. Harm Reduct J.

[21] Al-Lawati JA, Muula AS, Hilmi SA, Rudatsikira E (2008). Prevalence and determinants of Waterpipe tobacco use among adolescents in Oman. Sultan Qaboos Univ Med J.

[22] Dib JE, Haddad C, Sacre H, Akel M, Salameh P, Obeid S, Hallit S (2021). Factors associated with problematic internet use among a large sample of Lebanese adolescents. BMC Pediatr.

[23] Malaeb D, Awad E, Haddad C, Salameh P, Sacre H, Akel M, Soufia M, Hallit R, Obeid S, Hallit S (2020). Bullying victimization among Lebanese adolescents: the role of child abuse, internet addiction, social phobia and depression and validation of the Illinois bully scale. BMC Pediatr.

[24] Chahine M, Salameh P, Haddad C, Sacre H, Soufia M, Akel M, Obeid S, Hallit R, Hallit S (2020). Suicidal ideation among Lebanese adolescents: scale validation, prevalence and correlates. BMC Psychiatry.

[25] Hallit J, Salameh P, Haddad C, Sacre H, Soufia M, Akel M, Obeid S, Hallit R, Hallit S (2020). Validation of the AUDIT scale and factors associated with alcohol use disorder in adolescents: results of a National Lebanese Study. BMC Pediatr.

[26] Awad E, Haddad C, Sacre H, Hallit R, Soufia M, Salameh P, Obeid S, Hallit S (2021). Correlates of bullying perpetration among Lebanese adolescents: a national study. BMC Pediatr.

[27] Merhy G, Azzi V, Salameh P, Obeid S, Hallit S (2021). Anxiety among Lebanese adolescents: scale validation and correlates. BMC Pediatr.

[28] Comrey AL, Lee HB (2013). A first course in factor analysis.

[29] Maziak W, Ben Taleb Z, Jawad M, Afifi R, Nakkash R, Akl EA, Ward KD, Salloum RG, Barnett TE, Primack BA, Sherman S, Cobb CO, Sutfin EL, Eissenberg T, Expert Panel on Waterpipe Assessment in Epidemiological Studies (2017). Consensus statement on assessment of waterpipe smoking in epidemiological studies. Tob Control.

[30] Pokorny SB, Jason LA, Schoeny ME (2004). Current smoking among young adolescents: assessing school based contextual norms. Tob Control.

[31] Marsh HW, Hau K-T, Wen Z (2004). In search of golden rules: comment on hypothesis-testing approaches to setting cutoff values for fit indexes and dangers in overgeneralizing Hu and Bentler’s (1999) findings. Struct Equ Model.

[32] Breslau N, Fenn N, Peterson EL (1993). Early smoking initiation and nicotine dependence in a cohort of young adults. Drug Alcohol Depend.

[33] MacPherson L, Strong DR, Myers MG (2008). Using an item response model to examine the nicotine dependence construct as characterized by the HONC and the mFTQ among adolescent smokers. Addict Behav.

[34] Fernando WW, Wellman RJ, Difranza JR (2006). The relationship between level of cigarette consumption and latency to the onset of retrospectively reported withdrawal symptoms. Psychopharmacology.

[35] Rojas NL, Killen JD, Haydel KF, Robinson TN (1998). Nicotine dependence among adolescent smokers. Arch Pediatr Adolesc Med.

[36] Rose JS, Dierker LC (2010). An item response theory analysis of nicotine dependence symptoms in recent onset adolescent smokers. Drug Alcohol Depend.

[37] Maziak W, Rastam S, Shihadeh AL, Bazzi A, Ibrahim I, Zaatari GS, Ward KD, Eissenberg T (2011). Nicotine exposure in daily waterpipe smokers and its relation to puff topography. Addict Behav.

[38] Jawad M, Charide R, Waziry R, Darzi A, Ballout RA, Akl EA (2018). The prevalence and trends of waterpipe tobacco smoking: a systematic review. PLoS One.

